# Remote Assessment of Cultural Heritage Environments with Wireless Sensor Array Networks

**DOI:** 10.3390/s140508779

**Published:** 2014-05-19

**Authors:** Henoc Agbota, E. Mitchell John, Marianne Odlyha, Matija Strlič

**Affiliations:** 1 Centre for Sustainable Heritage, University College London, Gower St, London WC1E 6BT, UK; E-Mail: m.strlic@ucl.ac.uk; 2 Department of Electronic and Electrical Engineering, University College London, Gower St, London WC1E 6BT, UK; E-Mail: j.mitchell@ucl.ac.uk; 3 Birkbeck College, University of London, Malet Street, London WC1E 7HX, UK; E-Mail: m.odlyha@bbk.ac.uk

**Keywords:** WSN, piezoelectric quartz crystal, dosimeter, cultural heritage, developing countries, environmental monitoring, Royal Palaces of Abomey, Apsley House, Waspmote

## Abstract

The logistics and cost of environmental monitoring can represent challenges for heritage managers, partly because of the sheer number of environmental parameters to consider. There is a need for a system, capable of monitoring the holistic impact of the environment on cultural materials while remaining relatively easy to use and providing remote access. This paper describes a dosimetric system based on piezoelectric quartz crystal technology. The prototype sensing module consists of an array of piezoelectric quartz crystals (PQC) coated with different metals (Fe, Cu, Ni and Sn) and includes a temperature and relative humidity sensor. The communication module involves an 802.15.4 low-power radio and a GPRS gateway which allows real time visualisation of the measurements online. An energy management protocol ensures that the system consumes very low power between measurements. The paper also describes the results and experiences from two heritage field deployments, at Apsley House in London, UK, and at the Royal Palaces of Abomey in Benin. Evaluation of PQC measurements, temperature, relative humidity and the rate of successful transmission over the communication systems are also reported.

## 1. Introduction

Environmental monitoring is a key component of heritage management. Individual environmental parameters such as temperature, relative humidity and pollutants have been shown in laboratory experiments to cause damage to cultural materials. However, in the real environment, these parameters tend to act synergistically. For example, the synergistic action of heat and relative humidity and certain pollutants can cause or exacerbate loss of colour in photographic films, corrosion in metals and fading in textiles [1–3]. Most heritage sites systematically monitor temperature and relative humidity [4,5]. However, other environmental parameters such as pollutants, are harder to measure not only because the measurement cost of a single pollutant is generally higher than that of temperature and relative humidity combined but also because it is often necessary to measure multiple pollutants [5].

Conservators have long learned to holistically assess the impact of the environment on their objects and collections from simple visual inspections. In other terms, they use the actual materials as dosimetric sensors. This approach was previously tested using “mock paintings” consisting of differently pigmented paint strips which provided information concerning the synergistic action of the micro-climates surrounding paintings [6]. The dosimetric approach which measures the synergistic and accumulative effects of different factors on a given material has led to the development of early warning systems such as the recent MEMORI dosimeter [7].

Most current dosimeters typically consist of a single or a couple of materials known to be sensitive to a class of pollutants [8]. There is a need for more versatile systems which use an array of materials. Some dosimetric technologies are already capable of interfacing with several materials, more precisely metals [9]. However, a different device needs to be purchased for every metal of interest. There is therefore a need for dosimetric instrumentation to be able to flexibly interface different types of materials.

It is required for any instrumentation deployed in heritage environments to be as non-intrusive as possible. Recent advances in information and communication technologies have led to the development of small, self-powered devices capable of internally saving data as well as transmitting them remotely by radio [10]. These devices provide other benefits beyond their low aesthetic impact. They can be deployed in large numbers to provide high resolution with little additional overhead due to the fact that data acquisition is done remotely [11]. They provide *in situ* and localised measurements as they can be deployed in confined areas and historic buildings [12,13].

The paper describes an environmental monitoring system based on a piezoelectric quartz crystal (PQC) dosimeter array. In previous work with PQC crystals two different coatings (organic and inorganic) were used for monitoring indoor environments [14–16]. These showed a differential response. The organic coating based on artists' varnish responded to the oxidizing action of inorganic pollutants (NO_x_) and light. The inorganic coating (lead) responded to the presence of organic acids in display cases or microclimate frames for paintings. In this paper different metal coatings have been chosen for their known differential action to pollutants. In addition diffusive passive sampling tubes were included in the field tests to calibrate the response of the coated PQC crystals. The environmental impact on the coated PQC crystals is assessed from measured shifts in the oscillation frequencies of the crystals. This paper also describes the communication system which allows the remote transmission and real-time visualisation of the measurements online. The issues encountered during the deployments at the two case study sites in London and Abomey, as well as the system characterisation and performance evaluation results are also reported.

To the best of our knowledge, the innovations described in this paper are the following: (i) the dosimetric system is the first such unit capable of interfacing up to eight, not pre-determined, types of materials; (ii) the system makes it easier to remotely monitor several different environments that are representative of the actual intended use, including challenging environments such as outdoor and remote locations; (iii) the sensor responses (converted in percentages of coating mass increase) and the analysis of their correlation with environmental parameters, such as temperature, relative humidity and pollutant concentration, have helped characterise and compare the environments at two significant heritage sites in Europe and Africa.

## 2. System Architecture

In typical wireless sensor networks (WSN), little processing is done at the sensor node. Usually, the sensor node would be a single sensor whose sole task would be to transmit its measurements to a sink or gateway node. In this system a sensor array network approach was adopted in which data from an array of sensors, which includes temperature (T) and relative humidity (RH) sensor and coated PQC crystals, would be first aggregated at the sensor node before being transmitted to the gateway.

The main benefit of reducing radio communication in this way is to generate significant energy savings which would allow the system to operate autonomously over a long period. Indeed, radio communication is by far the most energy consuming component of WSN. For example, it could take less energy for some microcontrollers to run 100 instructions than to transmit a single bit of data [17]. A drawback of this approach, however, is a higher data load at the sensor node and hence a higher risk of significant data loss, particularly in applications where a node would fail and cannot be recovered, such as in some very harsh environments [10].

**Figure 1.**
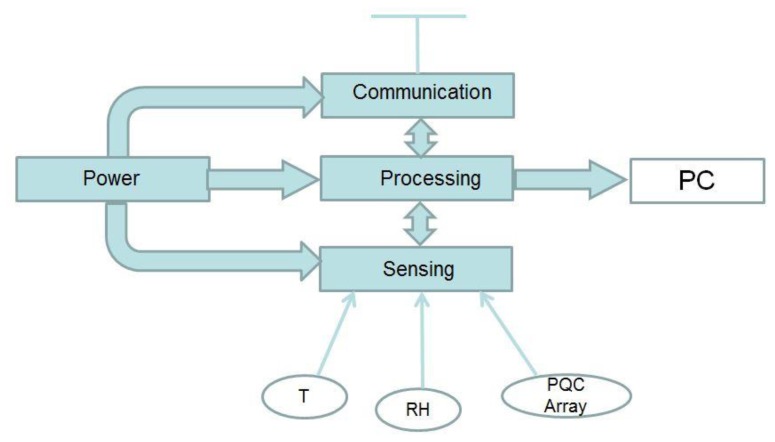
System architecture.

**Figure 2.**
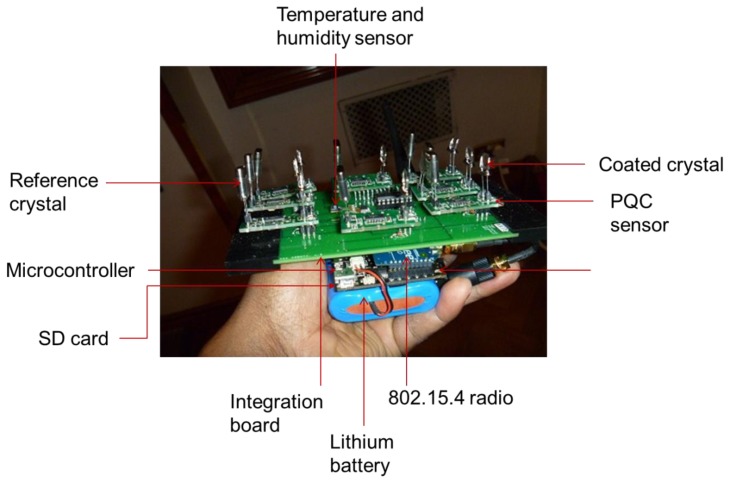
The PQC sensor array prototype.

The four main modules of the system architecture, sensing, processing and memory, communication systems and power management, are similar to traditional WSN architecture and are illustrated in Figure 1. They are described in detail in the following paragraphs. A photograph of the PQC sensor array prototype is shown in Figure 2. A smaller more integrated version is being developed to fit into confined spaces such as display frames.

### 2.1. Sensing

The sensing module is composed of a dosimeter array and a Sensirion SHT75 temperature and relative humidity sensor [18]. The dosimeter array consists of up to eight PQC crystals where each reference and coated crystal has its own oscillator driver circuit board custom-made by Quartz Technology Ltd [19]. The principle is that the environmental effect on the material will cause nanoscopic changes in its weight. For elastic materials, the frequency shifts of the coated PQC crystals can be related to the weight change of the coatings by the Sauerbrey equation [20]:

$$\Delta_{f}=-\frac{2f_{0}^{2}}{A\sqrt{\rho_{q}\mu_{q}}}\Delta_{m}$$

where f_0_: Resonant frequency (Hz); Δ_f_: Frequency change (Hz); Δ_m_: Mass change (g); A: Piezoelectrically active crystal area (gold coated area between electrodes, cm^2^); ρ_q_: Density of quartz (2.648 g/cm^3^) and μ_q_: Shear modulus of quartz for AT-cut crystal (2.947 × 1011 g/cm·s^2^). The PQC array consisted of copper (Cu), iron (Fe), nickel (Ni) and tin (Sn) coated PQC crystals prepared with three different thicknesses. These four metals were selected because of their presence in the cultural objects and collections of our case study heritage sites. These metals were also selected because of previous work which demonstrated their different sensitivities to inorganic pollutants, temperature and relative humidity [21–23]. Thus, in the long term, it might be possible to characterise the presence of these pollutants from the PQC sensor array response. Finally, the selected metals form elastic films which are amenable for use with PQC crystals. The films were coated on both sides of the crystals using high purity metals deposited by thermal evaporation. During deposition, the film thickness levels were monitored using an Edwards FTM2 rate monitor connected to a commercial 6 MHz quartz crystal. The targeted thicknesses were 50, 100 and 150 nm. However, as discussed in Section 4.3, in practice the deposited thickness can vary greatly.

### 2.2. Processing and Memory

The sensing unit is connected to a Waspmote, which is a wireless sensor network platform developed by Libelium (Zaragoza, Spain) and based on the open source Arduino language [24]. It is embedded with an 8-MHz Atmega 1281 microcontroller [25] and an 802.15.4, 2.4 GHz Xbee low-power radio [26]. The microcontroller multiplexes the eight PQC sensor signals. The PQC sensor outputs a transistor-transistor logic (TTL) signal corresponding to the frequency difference between a reference uncoated and unexposed crystal and an exposed crystal coated with the material of interest. For each PQC sensor, the frequency difference between coated and reference crystals is calculated by counting the rising edges of the TTL output signal. The Waspmote platform is embedded with a real time clock and a Secure Digital (SD) card which are used to timestamp the temperature, humidity and PQC measurements. Respectively. and save them locally.

### 2.3. Communication System

**Figure 3.**
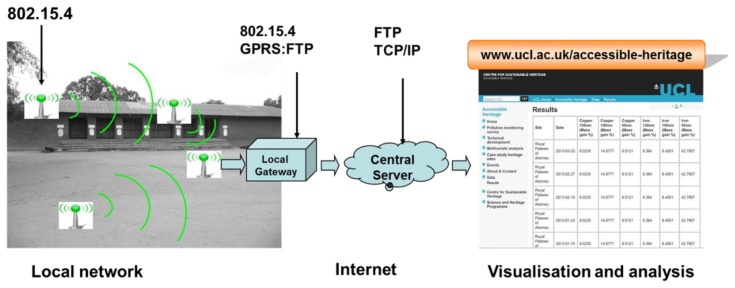
The communication system.

The communication system is designed to enable remote operation and is described in Figure 3. The PQC, temperature and relative humidity measurements are aggregated with other data, such as the measurement id, the device id and the remaining battery level, into the 128-byte payload of a 802.15.4 radio packet. The embedded Xbee radio hardware uses a carrier sense media access control protocol to transmit the data to a gateway device located within its 100-mW range at the heritage site.

The gateway is a mains powered Waspmote device which is continuously in receiver mode. The gateway is also equipped with a Simcom SIM900 General Packet Radio Service (GPRS)/Global System for Mobile Communications (GSM) module [27]. New measurements are appended to an internal file on the sensing device and transmitted to the gateway in an 802.15.4 radio packet. When the packet is received, the gateway stamps it with the current date and time and runs a file transfer protocol (FTP) over the GPRS connection to upload the measurements to a MySQL database on a Linux Apache MySQL PHP (LAMP) internet server. The FTP's handshake and data transfer are executed with GPRS AT commands. The main reason for the choice of FTP, compared to other protocols such as Transmission Control Protocol (TCP), Simple Mail Transfer Protocol (SMTP) and Wi-Fi which could also be used to remotely retrieve the measurements over GPRS, is to ensure an additional layer of backup of the data. Hence, the measurements are stored as files on the device and on the server and also as records on the database. PHP scripts are used to query the database and display the most recent measurements on the research project website in real time (Figure 3).

### 2.4. Power Management

In order to enable long-term autonomous operation, a power management protocol has been implemented. It consists of putting the device in a “hibernation” state in which all the hardware modules are switched off except the real time clock (RTC). The Waspmote is equipped with a lithium-ion battery 6600-mAh battery as well as a cell coin battery. In the hibernation state, the device is solely powered by the coin battery which provides a 0.7-μA current. The coin battery serves to power the 32-kHz DS3231 RTC [28]. The DS3231 is an Inter-Integrated Circuit (I^2^C) RTC with an integrated temperature-compensated crystal oscillator (TCXO) and crystal. The RTC is programmed to generate an alarm interrupt to “wake up” the device in time for the next series of measurement sampling. The measurement sampling rate which dictates the setting of the alarm clock can be varied from 8 s to minutes, hours and days. It is evident that the lower the sampling rates the lower the power consumption.

## 3. Deployment

### 3.1. Case Study Heritage Sites

The system has been deployed at two heritage sites: Apsley House in London and the Royal Palaces of Abomey in Benin. Apsley House, now an English Heritage property, is known as Number One London and was home to the Duke of Wellington. Apsley House is home to a celebrated art collection, which includes paintings initially from the Spanish Royal Collection. It also contains diverse objects brought by the Duke of Wellington from his war campaigns, including porcelain plates and vases, and silver-gilt centrepieces.

The Royal Palaces of Abomey are a UNESCO World Heritage site. The site consists of twelve palaces successively built by different sovereigns of the West African kingdom of Dahomey, from 1625 to 1900, in their capital Abomey. The collection includes royal thrones, weapons, jewels, textiles but also diverse objects imported from Europe. The site is perhaps most known for its rich set of bas-reliefs which report on events, beliefs and culture in Dahomey.

### 3.2. Experimental Description and Practical Issues

**Figure 4.**
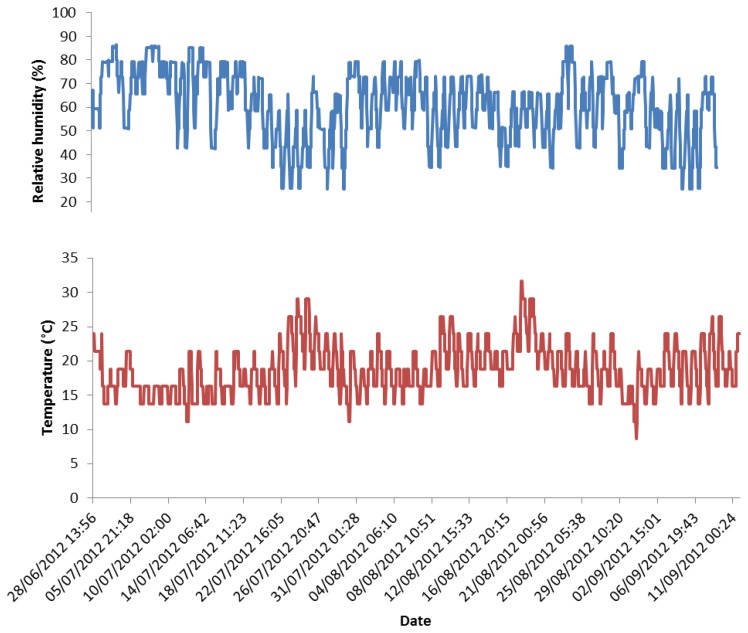
Outdoor hourly T and RH data from Apsley House from the end of June to early September 2012.

The system was initially deployed at Apsley House without the PQC sensors and the communication system. Figure 4 shows the temperature (T) and relative humidity (RH) data gathered in the first three months of deployment by the Sensirion SHT75 temperature and relative humidity sensor. The large variations in T and RH reflect the fact that the system was exposed outdoors. The Sensirion SHT75 is a commercially available sensor and its measurements have been validated outdoors against other climate monitoring instruments [29]. During the deployments, ozone (O_3_), nitrogen dioxide (NO_2_) and sulfur dioxide (SO_2_) levels were also measured with diffusion tubes.

Progressively, different features were added until the full system was in place. The full system consisted of two sensing devices and a gateway. The sensing devices each have a 6-PQC sensor array and all the features described in Section 2, including the communication system. The devices were housed in aluminum weather-proof boxes with a single opening covered by a polyethylene air particle filter. The chemical attenuation of the filter was measured to be 35%. It was calculated as the decrease in ozone concentration measured by diffusion tubes placed outside and inside the box. An hourly sampling rate was set. The same system was deployed at Abomey.

Different issues were encountered during the two deployments. At Apsley House, the key issue was the fact that the thickness of the walls made it difficult to find installation locations which allow for good radio communication between sensing devices and gateway on one hand as well as a good 3G coverage for the gateway on the other hand. At Abomey, frequent power outages interfered with the operation of the mains powered gateway.

## 4. Results

### 4.1. Laboratory Validation

Following the exposure the coated PQC crystals were brought to the laboratory for measurement to test the accuracy of recorded values. From the system the crystal frequency outputs were read. These were already subtracted from the uncoated reference crystals. To check these values, measurements were also conducted using the laboratory frequency measuring system where individual crystal frequencies and reference crystals were measured and their values checked against those obtained from the system. The laboratory conditions were measured at 21 °C and 34% relative humidity. The laboratory instrument is the high-accuracy EIP model 578 source locking continuous wave microwave frequency counter with selective power measurement [30].

**Figure 5.**
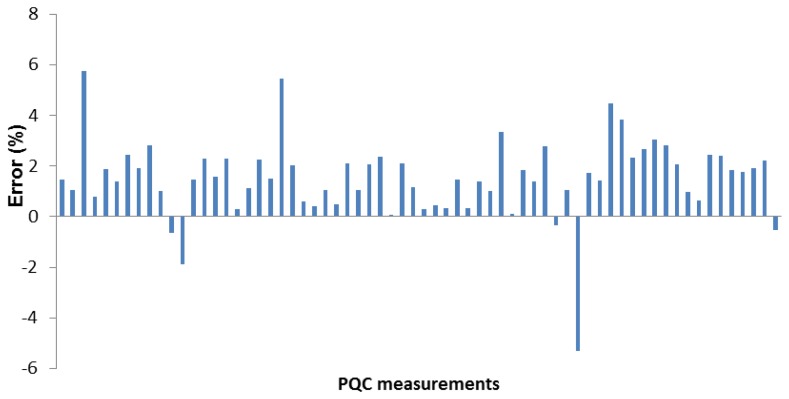
Frequency measurement errors (%) between the sensor array device and the EIP 578 instrument in laboratory conditions.

Accuracy is defined here as the percentage error between the PQC measurements of our dosimetric sensor array device and values recorded for the individual crystals when measured with the EIP 578 frequency counter. The results of 69 measurements are reported in Figure 5. The average error is 1.5% (standard deviation 1.6).

Noise from the microcontroller's 10-bit analog-to-digital converter (ADC) probably contributes to the observed error. The error is also likely due to the mechanical stress caused by the handling of the crystals during the test, even though great care was taken. Indeed, by the principle of piezo-electricity, mechanical stress will cause a voltage across the crystal which will conversely cause it to begin to vibrate.

### 4.2. Delivery Rate

Delivery rate is defined as the percentage of radio packets received at the Internet server out of the total number of packets sent by a sensing device. It is straightforward to calculate since every measurement packet is incrementally numbered by the sensing device.

**Figure 6.**
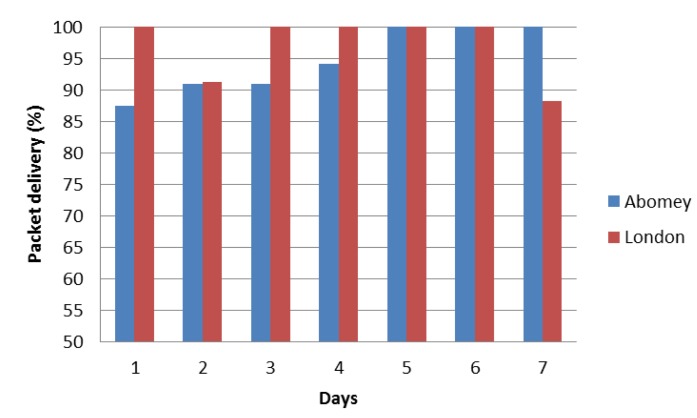
Daily averaged delivery rate of the communication system over a week.

Figure 6 shows the daily averaged delivery rates at Abomey and London over a week period. For Abomey, this period was the first week of deployment. During this period, we were able to record the time periods of power cuts and exclude them from the calculations. The figure shows that the communication system delivers over 85% of the measurements at both locations. A 100% daily delivery rate was achieved three times in a week at Abomey and four times in London.

The fact that the delivery rate at Abomey is lower than London for four days a week is probably due to better mobile network coverage in London. However, it should be noted that the data for Abomey does not include power cut periods. The recorded accumulative power cut period over that week was 12 h.

### 4.3. PQC Measurements

More detailed analysis of the PQC data is ongoing and will be published in the future. Tables 1 and 2, however, show how it can be used to assess the impact of different environments on heritage materials. Our analysis here focuses on a comparison of the percentage of mass increase between the two locations and the relation between the coating thickness and the percentage of mass increase.

Regarding the relation between the percentage of mass increase and the coating thickness, with two exceptions, the 50-nm sensors consistently show the highest mass increase. The two exceptions occured in Abomey with Cu and Sn, where, as previously mentioned, the corrosion rates of these two metals might be too low to be accurately measured with these thickness levels. Other dosimetric sensors have reported similar relation between sensor thickness and sensitivity [9]. The results also show that there is no clear difference in sensitivity for the highest thickness levels of 100 nm and 150 nm. Indeed, in half of all the cases, 150 nm sensors have measured higher corrosion rate than 100 nm sensors.

**Figure 7.**
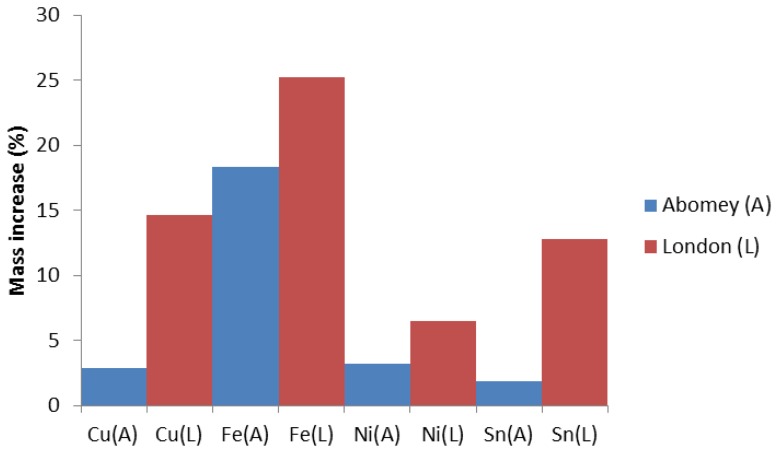
Percentage of mass increase measured by 50 nm PQC sensors in London (L) and Abomey (A) over 140 days between November 2012 and April 2013.

**Figure 8.**
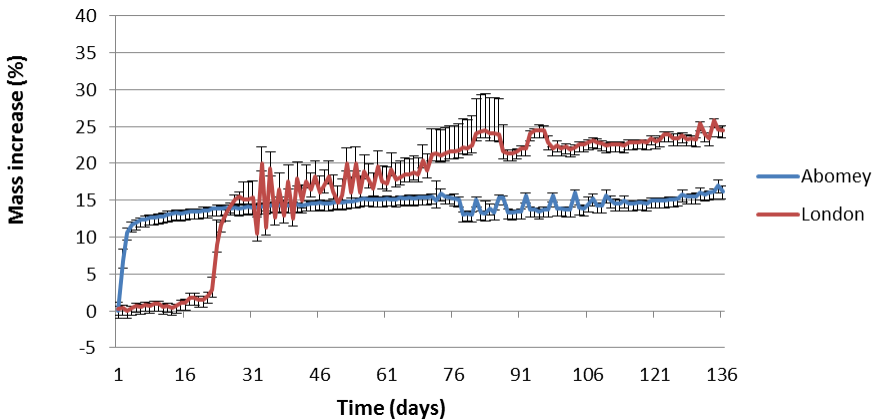
Daily averaged percentage of mass increase of 50-nm Fe sensors in Abomey and London during 140 days of exposure between November 2012 and April 2013 with standard deviations as error bars.

We will therefore now focus on the more sensitive 50 nm sensors. As shown in Figure 7, they have all reported higher corrosion rates in London than Abomey. The results can be explained by the higher humidity and overall pollution levels (expressed in parts per billion or ppb) in London as shown in Table 2. Indeed, other than O_3_, which is higher in Abomey, RH and NO_2_ are all significantly higher in London while SO_2_ is negligible at both locations. The results confirm previous literature which has shown that these parameters are major factors in the corrosion of Cu, Fe, Ni and Sn [21–23]. The initial slower response of the Fe sensor in London (Figure 8) is probably explained by the lower temperature (6.1 °C average temp. November-April 2013) compared to Abomey (27.4 °C average temp. same period). Indeed, as with most chemical reactions, iron corrosion would proceed more rapidly in a warmer environment.

**Figure 9.**
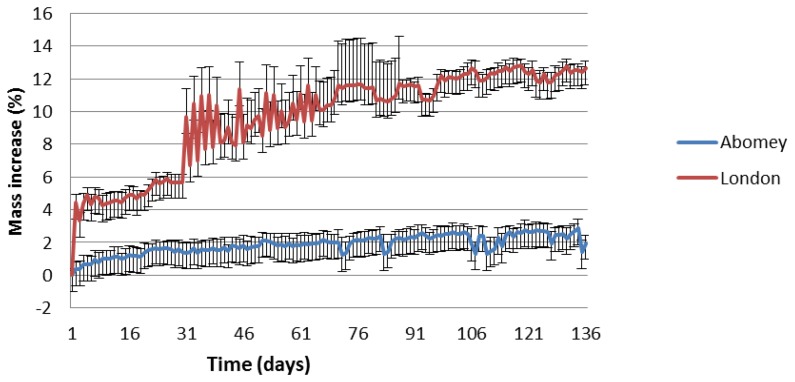
Daily averaged percentage of mass increaseof 50-nm Cu sensors in Abomey and London during a period of 140 days of exposure between November 2012 and April 2013 with standard deviations as error bars.

Figure 8 illustrates that corrosion is non-linear and that the result of the comparative assessment of how corrosive two different environments are can vary over time. The 50-nm Fe percentage of mass increase was initially faster and higher in Abomey than Apsley House. However, after 27 days, the two plots cross and the corrosion rate progressively becomes higher in London and stable in Abomey. After 140 days, the sensor response is 10% higher in London. In contrast, for Cu, the corrosion rate was from the start higher in London than Abomey, as shown in Figure 9. After 140 days, the 50-nm Cu percentage of mass increase is five times higher in London.

Temperature changes are known to cause frequency changes in crystals. Exposure under outdoor conditions, where temperature is variable, can cause rapid fluctuations in crystal frequency, as seen in Figures 8 and 9. Furthermore, the parameters of the Sauerbrey equation, such as the shear modulus of quartz for AT-cut crystals, have been derived for relatively stable room temperature conditions. The accuracy of the system is therefore expected to be worse in outdoor conditions. As future work, we plan to address this problem by implementing a temperature compensation algorithm.

## 5. Discussion and Future Work

The system described in this paper has the ability to assess both organic and inorganic materials. For example, varnish and mastic coated PQC crystals have already been successfully used in previous research to evaluate the corrosivity of the microclimates in the vicinity of paintings [6]. The fact that the same instrument can be used with an array of different materials and the coated PQC crystals can be exchanged makes it very versatile in comparison to existing dosimetric systems. In addition, the communication system and energy efficient performance allow autonomous operation and remote data logging, hence making the system suitable for different environments, indoor and outdoor.

In future, collecting more PQC and environmental data should improve the interpretation of the system results by making it possible to compare the impacts of more environments on different materials. The PQC array responses could also be analysed against the environmental parameters expected to have a changing effect on the coated materials, such as temperature, humidity and pollutants. This could help derive, for example, whether a given environment is likely to have a higher concentration of certain pollutants.

## 6. Conclusions/Outlook

The paper describes an environmental monitoring prototype designed to assess the impact of the environment on cultural heritage. The prototype consists of an array of piezoelectric quartz crystals and a temperature and relative humidity sensor. The measurements can be both logged on the device or uploaded remotely to an internet server and visualised in real-time.

The average percentage error of the system is shown to be 1.8% compared to a high precision laboratory analytical instrument. The system was deployed at Apsley House in London, a property of English Heritage and at the Royal Palaces of Abomey in Benin, a UNESCO World Heritage site. The system performance during these deployments is reported. The communication system is shown to deliver over 85% of the measurements both in Abomey and London.

The PQC measurements show that the impact of the environment on copper and iron materials is more damaging in Apsley House than Abomey. It was found that the thinnest PQC film (50 nm) is more sensitive and responds fastest to the environment. The results suggest that thinner sensors would be suitable for quick environment assessment applications while thicker sensors would be ideal for long-term environment monitoring applications.

Key assets of the system include its great versatility. It can be used to assess up to eight different materials and is not designed for a particular material or type of materials. Its autonomous operation and remote data logging features make it suitable and adapted to different environments. Other applications of the system could exist outside heritage environment and material monitoring. For example, construction or oil and gas companies could be interested in an early warning systems which provide them with real-time remote testing of different materials.

## Figures and Tables

**Table 1.** Percentage of mass increase measured by PQC sensors in London (L) and Abomey (A) over 140 days between November 2012 and April 2013.

**Coating**	**Thickness (nm) (A)**	**Thickness (nm) (L)**	**% Mass Increase (A)**	**% Mass Increase (L)**
Cu	48	42	2.9	14.6
Cu	98	94	4.0	2.4
Cu	132	125	1.6	1.6
Fe	63	53	18.3	25.2
Fe	96	82	5.7	7.5
Fe	138	143	6.7	16.4
Ni	46	43	3.2	6.5
Ni	86	87	0.9	2.7
Ni	129	178	1.5	0.8
Sn	36	39	1.9	12.8
Sn	84	77	2.6	7.1
Sn	104	109	3.4	1.0

**Table 2.** Average temperature, relative humidity and pollution levels measured in London and Abomey over 140 days between November 2012 and April 2013.

**Location**	**T (°C)**	**RH (%)**	**O_3_ (ppb)**	**NO_2_ (ppb)**	**SO_2_ (ppb)**
Abomey	27.4	67.5	43.2	4.1	1.0
London	6.1	79.2	16.0	17.4	1.8
